# Salbutamol and Formoterol Attenuate Okadaic Acid-Induced Cytotoxicity in Undifferentiated PC-12 Cells: Evidence for a β2-Adrenergic Receptor-Independent Protective Component

**DOI:** 10.3390/cimb48060571

**Published:** 2026-05-29

**Authors:** Andria Kotsoni, Evelina Ioannou, Evangelia Barmparousi, Lefteris C. Zacharia

**Affiliations:** 1 Department of Health Sciences, School of Life and Health Sciences, University of Nicosia, 46 Makedonitissas Ave, CY-2417 Nicosia, Cyprus; andriakins99@hotmail.com (A.K.); ioannou.e30@live.unic.ac.cy (E.I.); barmparousi.i@live.unic.ac.cy (E.B.); 2 Bioactive Molecules Research Center, School of Life and Health Sciences, University of Nicosia, 46 Makedonitissas Ave, CY-2417 Nicosia, Cyprus

**Keywords:** β2-adrenergic receptors, formoterol, salbutamol, PC-12 cells, okadaic acid, okadaic acid-induced cytotoxicity

## Abstract

β2-adrenergic receptor (β2-AR) agonists have been implicated in neuroprotection, yet their mechanisms remain obscure. We examined whether salbutamol (SA, short-acting) or formoterol (FO, long-acting) protect PC-12 cells from okadaic acid (OA), and evaluated receptor dependence, antioxidant capacity, and apoptotic signaling. Viability was quantified with crystal violet and MTT assays. OA reduced viability to approximately 60%, and SA or FO (0.1–10 µM) improved survival, which reached 76–83% at 10 µM (*p* < 0.05). β2-AR blockage with ICI-118,551, and *ADRB2* mRNA knockdown did not abolish protection by FO or SA, suggesting a possible β2-AR-independent protective component. However, as knockdown was not confirmed at the protein level and signaling was not directly assessed, the evidence remains provisional. FO, but not SA, exhibited direct antioxidant activity in the DPPH assay, but both at 50 μΜ lowered H_2_O_2_-induced intracellular reactive oxygen species (from 167.9% to baseline, *p* < 0.05). Both compounds reduced the OA-induced expression of selected pro-apoptotic transcripts, although these mRNA data do not establish the functional inhibition of apoptosis. FO reduced fold change relative to untreated control from 5.8 to 2.6 for *Bax*, and 6.4 to 3.4 for *Bak*, whereas SA achieved a significant reduction only for *Bax*, from 5.8 to 4.4. Taken together, SA and FO offer a partial protection to PC-12 cells from OA cytotoxicity through pathways suggesting a β2-AR-independent protective component, with FO showing additional antioxidant properties and a reduced expression of selected pro-apoptotic transcripts. These findings provide preliminary evidence that select β2 agonists may exert cytoprotective effects that are consistent with, but do not establish, a receptor-independent component. These findings warrant further protein-level and functional validation.

## 1. Introduction

Neurodegenerative diseases, such as Alzheimer’s disease (AD), are characterized by irreversible neuron loss and severe, life-threatening symptoms. AD is the most prevalent neurodegenerative disorder, marked by progressive cognitive impairment and memory loss, and eventually leading to the loss of motor control and a vegetative state until death [1,2]. The disease is characterized by the formation of amyloid plaques due to beta-amyloid peptide (Aβ) deposition, microglial immune responses, and neurofibrillary tangles composed of hyperphosphorylated tau protein, leading to neuronal death and synaptic loss [2,3]. Despite extensive drug development efforts, there remains an unmet need for disease-modifying treatments for AD. Furthermore, drug repurposing has received increasing attention as a strategy for identifying compounds with potential activity in neurodegeneration-related models; adrenergic receptor-modulating drugs are of interest in this context, although the literature remains mixed.

Several studies support protective effects of β2-adrenergic receptor (β2-AR) activation, including improved synaptic plasticity, reduced inflammatory signaling, and protection from Aβ-related impairment [4,5,6,7,8]. In contrast, other studies suggest that abnormal β2-AR activation may increase γ-secretase activity, promote amyloid pathology, or contribute to AD-related processes under certain conditions [9].

Specifically, the role of asthma drugs acting on β2-AR has been studied more extensively in vivo, with mixed results. For example, the β2 agonist formoterol has been shown to improve synaptic plasticity and cognitive function in a mouse model of Down syndrome [4]. The activation of β2-ARs, but not of β1, prevents the Aβ-evoked inhibition of long-term potentiation (LTP) in adult animals [5]. Other studies have shown that β-antagonists and β2-agonists can improve memory and reduce brain damage in rats [10]. A recent cohort study suggested that selective β2-AR agonists are associated with a decreased risk of developing AD, while non-selective AR antagonists are associated with an increased risk [6]. Additionally, β2-AR activation has been linked to decreased neuroinflammation and improved cognition in AD models [7,8]. In an in vitro study, dobutamine and salbutamol reduced both the rate and yield of tau-filament formation, but only salbutamol additionally inhibited tau’s structural change into β-sheet-rich aggregates [11]. However, some studies indicate that abnormal β2-AR activation might contribute to Aβ accumulation and AD pathogenesis [9]. Several reviews have summarized both protective and potentially detrimental roles of adrenergic signaling in neurodegeneration-related models [12,13].

Despite evidence that β2-agonists can influence neurodegeneration-related endpoints in some models, it remains unclear whether such effects require β2-AR activation or reflect receptor-independent properties of the compounds. To better understand the role of β2-AR in neuroprotection and neurotoxicity, we tested salbutamol and formoterol (long-acting and short-acting β2-AR agonists, respectively) for their protective effects in PC-12 cells. Specifically, we investigated whether they protect PC-12 cells against okadaic acid-induced cell death. Okadaic acid (OA), a phosphatase inhibitor, is known to cause neurodegeneration, tau hyperphosphorylation, and oxidative stress, and OA-induced neurotoxicity is used to study AD pathology [14]. Although OA is useful for studying selected cytotoxic processes, it represents an acute pharmacological insult and does not reproduce the full complexity of AD. Therefore, findings from OA-based models should be interpreted as mechanistic in vitro observations rather than direct evidence of disease modification.

We aimed to test the hypothesis using two different viability assays: the MTT and the crystal violet assays. Although both assays are commonly used for evaluating cell viability, they demonstrate different cellular attributes. The MTT assay measures mitochondrial metabolic activity, while the crystal violet assay assesses the number of adherent cells, thus providing an estimate of total cell biomass. Utilizing both assays allowed us to gain a more comprehensive understanding of the protective effects of both drugs from the metabolic and structural perspectives. To further study the mechanism, we pharmacologically blocked the β2-AR, or silenced it using siRNA. Finally, in an effort to shed light on the possible mechanism of protection we studied the antioxidant potential of the test articles as well as their effect on pro-apoptotic genes.

## 2. Materials and Methods

### 2.1. Cell Culture

Rat pheochromocytoma cell line PC-12 (ATCC, Manassas, VA, USA) was grown in Ham’s F12K (Kaighn’s) medium supplemented with 15% horse serum (HS), 10% fetal bovine serum (FBS), and 1% penicillin/streptomycin, and incubated in a CO_2_ incubator at 37 °C. Cells were cultured to 70–80% confluence in 25 cm^2^ cell culture flasks and trypsin-EDTA (0.25%) was used for the detachment of cells. PC-12 cells were used in the undifferentiated state. No nerve growth factor (NGF)-induced differentiation was performed before drug treatment. Accordingly, the findings should be interpreted as data from pheochromocytoma-derived PC-12 cells rather than mature neurons.

### 2.2. Cell Viability Assays

3-(4,5-Dimethylthiazol-2-yl)-2,5-Diphenyltetrazolium Bromide (MTT) Assay. Cells were seeded in flat-bottom 96-well plates at a density of 1 × 10^4^ cells/well. Once reaching confluency, 24 h after seeding, cells were exposed to various concentrations of formoterol and salbutamol (0.1–1 μM), as well as a single concentration of ICI-118,551 (hydrochloride, >98% purity, Sigma Aldrich, St. Louis, MO, USA,100 nM) and okadaic acid (OA, ammonium salt from Prorocentrum concavum, >90% purity, Sigma Aldrich, St Louis, MO, USA, 50 nM) for 24 h. Formoterol (as fumarate dihydrate, >98% purity, Sigma Aldrich, St Louis, MO USA), and salbutamol (Sigma Aldrich, St Louis, MO, USA) were dissolved in DMSO, ensuring that the final solvent concentration never exceeded 0.1%. ICI-118,551 and OA were dissolved in distilled water.

All incubations with drugs were performed using media with low serum (2.5% FBS). In each experiment, cells treated with DMSO-only or untreated cells served as controls. MTT powder was dissolved in phosphate-buffered saline (PBS) at a concentration of 1 mg/mL. After treatment, 10 μL of the MTT solution was added to each well and incubated at 37 °C for 3 h. Finally, the medium was removed and 100 μL of DMSO was added to each well, all of which were then placed on a rocker for 20 min. The absorbance was measured at 570 nm with a Perkin Elmer Victor X3 2030 multi-mode microplate reader (Shelton, CT, USA). Three independent experiments were performed, each with at least 3 technical replicates.

### 2.3. Crystal Violet Staining for Cell Viability

Cells were seeded in flat-bottom 96-well plates at a density of 5 × 10^3^ cells/well overnight. Cells were then treated for 24 h with increasing concentrations of formoterol and salbutamol (0.1–10 μM), as well as a single concentration of ICI-118,551 (100 nΜ) and OA (50 nM). Treatments were conducted in medium supplemented with 2.5% FBS. Control conditions included untreated cells and cells exposed to DMSO alone. After a 24-h incubation with treatments, cells were fixed for 15 min with a methanol–ethanol (2:1) solution and washed with PBS. Cells were stained with crystal violet, and excess dye was removed by washing with distilled water. Retained cell-associated staining was used as an estimate of adherent cell biomass. Finally, cells were left to air-dry overnight, and the crystal violet was dissolved using an acetic acid solution. Absorbance was measured at 590 nm using a Perkin Elmer Victor X3 2030 multi-mode microplate reader. At least three independent experiments were performed with at least three technical replicates in each. The concentrations used in this study were selected for in vitro screening and mechanistic evaluation.

### 2.4. siRNA Transfection

PC-12 cells were seeded in flat-bottom 6-well plates at a density of 4 × 10^5^ cells/well, aiming to achieve approximately 60% confluency after 24 h of incubation. Cells were then transfected with negative control (Thermo Fisher, Waltham, MA, USA, Cat No. AM4635) or siRNA targeting *ADRB2* (AMBION, Austin, TX, USA, Cat No. AM16706), using the Lipofectamine 2000 reagent (Invitrogen Life Technologies, Carlsbad, CA, USA). Cells were harvested 24 h post-transfection and gene expression analysis followed to assess silencing efficiency by real-time PCR. For crystal violet-based cell viability assay, cells were trypsinized 24 h post-transfection, plated on 96-well plates and then treated with formoterol, salbutamol and OA for an additional 24 h.

### 2.5. RNA Isolation and Real-Time PCR

Total RNA was extracted and purified from PC-12 cells using the NucleoSpin RNA Kit (Macherey-Nagel, Düren, Germany) according to the manufacturer’s instructions. RNA concentration and purity were assessed using the IMPLEN nanophotometer. Pure RNA samples (with an A260/A280 ratio close to 2.00 but not lower than 1.80) were transcribed to cDNA using Primescript cDNA Synthesis Kit (Takara, Nojihigashi, Japan) and using 1 μg of RNA. The resulting cDNA was diluted 1:10 before the experiment proceeded to gene expression by real-time PCR. For the housekeeping gene, *β-actin* was used, and for each sample six replicates were run, three with the test primer and three with the *β-actin* primer. Each reaction had a total volume of 10 μL, which included 1 μL of cDNA, 1 μL of the respective primer mixture (forward and reverse primer, 10 μM each), 3 μL of dH_2_O and 5 μL SYBR green reagent (Kapa Biosystems, Wilmington, MA, USA). Replicates were placed in a 96-well PCR plate and the real-time PCR was performed using a LightCycler 480 instrument II from Roche (Rotkreuz, Switzerland). Gene expression levels of *ADRB2*, *BAX* and *BAK* were analyzed using gene-specific primers (Integrated DNA Technologies—IDT). The sequences of the primers used were as follows: *rADRB2* forward (F): 5′-GAG ACC CTG TGC GTG ATT GC-3′, *rADRB2* reverse (R): 5′-CCT GCT CCA CCT GGC TGA GG-3′, *rBAK* forward (F): 5′-AAT GGC ATC CGG ACA AGG AC-3′, *rBAK* reverse (R): 5′-TGT TCC TGC TGG TGG AGG TA-3′, *rBAX* forward (F): 5′-GGC GAT GAA CTG GAC AAC-3′, *rBAX* reverse (R): 5′-CCG AAG TAG GAA AGG AGG-3′, *β-actin* forward (F): 5′-AGG GAA ATC GTG CGT GAC AT-3′, *β-actin* reverse (R): 5′-AAC CGT TCA TTG CCG ATA GT -3′.

All genes were tested under the same conditions which included an initial 3 min incubation at 95 °C, followed by 35 cycles at 95 °C for 10 s, 60 °C for 30 s, and 72 °C for 30 s. Melting curve analysis was conducted after the completion of PCR to ensure a single peak was detected and amplified. At least 3 independent experiments were performed. Quantification of relative gene expression was performed using the ΔΔCt method.

### 2.6. 2′,7′-Dichlorodihydrofluorescein Diacetate (DCFDA) Assay

Intracellular reactive oxygen species (ROS) levels were measured using the fluorescent probe 2′,7′-dichlorofluorescin diacetate (DCFDA, Sigma-Aldrich, St. Louis, MO, USA). Cells were seeded in a 96-well flat-bottom plate and incubated with 100 μL of 25 μM DCFDA (prepared in phenol red-free medium) for 1 h at 37 °C. Following incubation, the DCFDA solution was removed, and the cells were washed with PBS. Subsequently, the cells were treated for 1 h with H_2_O_2_ (500 µM) alone or in combination with formoterol or salbutamol at 10 or 50 µM. Control conditions included DCFDA-only (25 μM), and a blank consisting of cells with medium-only. All treatments were performed in phenol red-free medium to minimize background fluorescence. Fluorescence intensity was measured at excitation/emission wavelengths of 485/535 nm using a Perkin Elmer Victor X3 2030 multi-mode microplate reader, after 1 h of incubation with the treatments. Values were blank-corrected and expressed relative to DCFDA-loaded untreated control cells, set as 100%. DCFDA fluorescence was interpreted as ROS-associated fluorescence rather than as a specific measure of a single reactive oxygen species. At least three independent experiments were performed, and each concentration was tested in sextuplicate.

### 2.7. 2,2-Diphenyl-1-Picrylhydrazyl (DPPH) Assay

The antioxidant potential of formoterol and salbutamol was evaluated using the DPPH free radical scavenging assay, with curcumin as a reference antioxidant. This assay was used as a cell-free measure of direct radical-scavenging activity. A DPPH stock solution was prepared by dissolving 1 mg of DPPH in 13 mL of methanol (0.2 mM); this was kept in the dark until use. For each reaction, 1500 μL of methanol was mixed with the test compound and 1500 μL of DPPH solution. Formoterol, salbutamol, and curcumin were tested at final concentrations of 1, 10, 25, 50, and 100 µM. The control sample contained only methanol and DPPH, without the test compound, while the blank consisted of methanol-only to calibrate the spectrophotometer. Mixtures were incubated in the dark at room temperature for 30 min. Absorbance was measured at 517 nm using the Jasco V-730 spectrophotometer (Tokyo, Japan). At least three independent experiments were performed, and each concentration was tested in triplicate.

For all assays, biological replicates were defined as independent experiments performed on separate days using separate cell passages. Technical replicates were replicate wells within the same experiment.

Formoterol and salbutamol were tested at 0.1, 1, and 10 µM in viability assays because these concentrations did not reduce viability when administered alone, and this allowed evaluation of concentration-associated protection against OA. The 50 µM concentration used in the DCFDA assay was included to assess whether the compounds could modulate H_2_O_2_-induced intracellular ROS-associated fluorescence under a stronger oxidative challenge. These concentrations were not intended to represent expected free CNS exposure in vivo.

### 2.8. Statistical Analysis

Statistical differences among groups were assessed using one-way ANOVA, followed by Tukey’s multiple-comparisons test for all pairwise group comparisons. Tukey’s procedure was used to control the family-wise type I error rate. A *t*-test was used where two groups were compared.

## 3. Results

**Figure 1:**
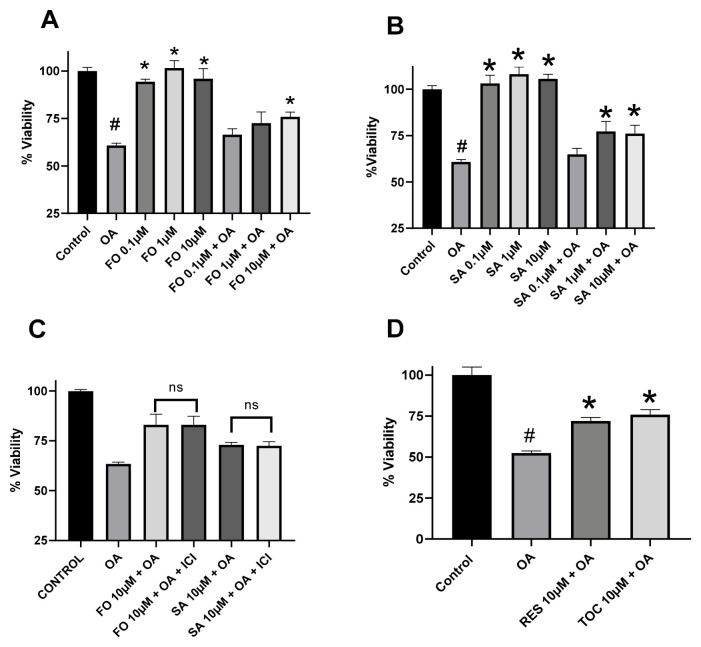
Protective effects of formoterol and salbutamol against OA-induced cytoxicity in PC-12 cells, assessed by crystal violet. Cell viability was assessed by crystal violet staining following treatment with (**A**) formoterol (FO) or (**B**) salbutamol (SA) at increasing concentrations (0.1, 1 and 10 μΜ), alone, or in combination with okadaic acid (OA, 50 nM) for 24 h. (**C**) Effect of ICI 118,551 (ICI), a β2-AR antagonist, on SA or FO protection from OA-induced cell death. (**D**) Effect of resveratrol (RES, 10 μΜ) or α-Tocopherol (TOC, 10 μΜ) on OA-induced cell death. Data are presented as mean ± SEM from *n* = 3 independent experiments, with at least 3 technical replicate wells per condition per experiment. Statistical analysis was performed using one-way ANOVA followed by Tukey’s multiple-comparison test. ^#^ *p* < 0.05 versus untreated control; * *p* < 0.05 versus OA; ns, not significant.

We first tested whether the selective β2-AR agonists formoterol and salbutamol attenuate OA-induced loss of viability in undifferentiated PC-12 cells. Cell-associated crystal violet staining was used as an estimate of adherent cell biomass after 24 h treatment. PC-12 cells were chosen as the model cell line usually employed to study neurodegeneration. Because the cells were not NGF-differentiated, the findings should be interpreted as results from undifferentiated PC-12 cells rather than mature neurons. OA at 50 nM induced ~40% cell death in PC-12 cells, as assessed by the crystal violet assay (*p* < 0.05 vs. control cells), while both salbutamol and formoterol protected the cells and reversed OA-induced cell death. As shown in Figure 1A, in the presence of OA viability was 60.8 ± 1.2%, whereas co-treatment with formoterol at 0.1, 1 and 10 μM increased viability to 66.5 ± 3.1%, 72.6 ± 5.8% and 75.9 ± 2.5%, respectively. The effect was statistically significant only with the 10 μM formoterol concentration, compared to OA-only treated cells, and there were no statistical differences between the three concentrations tested. A similar pattern was observed in the salbutamol-treated cells, where the viability was increased from 60.8% to 64.8 ± 3.3%, 77.3 ± 5.3%, and 76.1 ± 4.4% at 0.1, 1 and 10 μM salbutamol, respectively (Figure 1B). Statistical significance was observed only for the 1 and 10 μM salbutamol concentrations versus OA-treated cells, and there were no statistical differences among the three concentrations tested. In both experiments, salbutamol or formoterol alone had no effect on viability, while the protective effect was not statistically significant at the 0.1 μΜ concentration used. Interestingly, reversal was partial even with the 10 μM concentration used. Formal comparison between formoterol and salbutamol at matched concentrations did not reveal a significant difference in protection in the crystal violet assay. Finally, resveratrol and α-tocopherol, two known neuroprotective agents, were able to reverse OA-induced cell death. Specifically, viability with OA was 55.5 ± 1.6%, and in the presence of 10 μM resveratrol or α-tocopherol, viability increased to 68.7 ± 1.9% and 75.3 ± 1.8%, respectively (*p* < 0.05).

Despite not observing a full reversal effect at any tested concentrations, we further examined if the effects of the drugs were mediated by the β2-AR. The β2-AR was thus pharmacologically blocked using ICI-118,551 (ICI), a β2-selective blocker (Figure 1C). Interestingly, blockage of the β2 receptors with the selective inhibitor ICI did not reduce the protective effects of either FO or SA relative to OA-induced cell death. As shown in Figure 1C, viability with OA treatment was 63.5 ± 0.8%. The viability after treatment with 10 μM formoterol and OA was increased to 83.0 ± 5.3%, and in the presence of ICI, viability remained at 83.0 ± 4.3% (*p* < 0.05 v OA-treated cells). Interestingly, the same pattern was observed with salbutamol. Treatment with 10 μM salbutamol and OA increased viability from 63.5% (OA only) to 73.0 ± 1.2%, while in the presence of ICI viability remained at 72.5 ± 2.1% (*p* < 0.05 v OA-treated cells). Treatment of ICI with OA had no effect and remained at 63.1 ± 2.1%. Thus, under the present experimental conditions, protection was retained during pharmacological β2-AR blockage. However, because functional confirmation of β2-AR blockage was not performed, these data do not definitively exclude receptor involvement.

**Figure 2:**
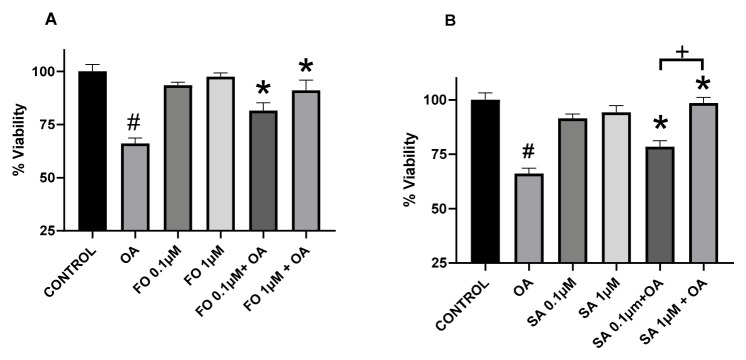
Effect of formoterol and salbutamol on OA-induced cytotoxicity in PC-12 cells, assessed by MTT assay. PC-12 cells were treated for 24 h with (**A**) formoterol (FO) or (**B**) salbutamol (SA) (0.1 and 1 μΜ), with or without OA (50 nM). Data are presented as mean ± SEM from *n* = 3 independent experiments, with 3 technical replicate wells per condition per experiment. MTT signal reflects mitochondrial-reducing activity, and is expressed relative to untreated control cells. Statistical analysis was performed using one-way ANOVA followed by Tukey’s multiple-comparison test. ^#^ *p* < 0.05 versus untreated control; * *p* < 0.05 versus OA. ^+^ *p* < 0.05.

Protection of OA-induced PC-12 cell death by FO and SA was also confirmed by the MTT assay, an alternative viability assay (Figure 2). As shown in Figure 2, 50 nM OA reduced viability to 66%, and formoterol at 0.1 and 1 μM reversed viability to 81.5 ± 3.8% and 91.0 ± 4.9%, respectively (*p* < 0.05 vs. OA). Similarly, salbutamol at 0.1 and 1 μM reversed viability from 66% to 78.4 ± 2.7% and 98.5 ± 2.7%, respectively (*p* < 0.05 vs. OA). Comparison between formoterol and salbutamol at matched concentrations did not reveal a significant difference in protection in this assay; however, the 1 μΜ SA + OA treatment was statistically different from the 0.1 μΜ SA + OA treatment. The MTT results are complementary to the crystal violet findings but should not be interpreted as an identical measure of viability. Crystal violet staining reflects adherent cell biomass, whereas MTT reduction reflects mitochondrial-reducing activity. Therefore, the stronger recovery observed in the MTT assay may indicate preservation of metabolic activity, assay-specific sensitivity, or both.

**Figure 3:**
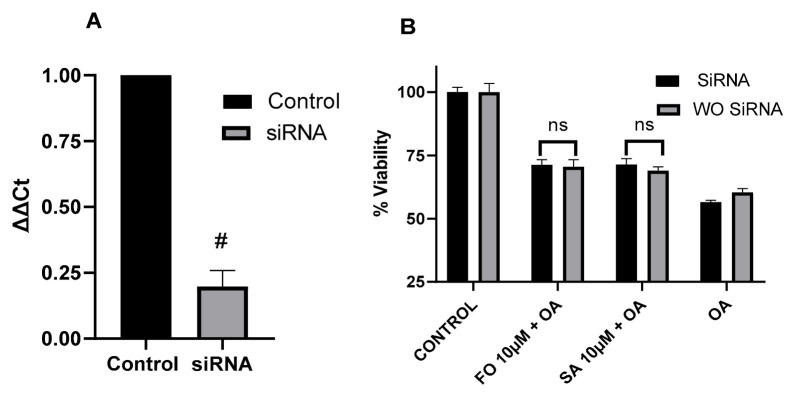
Silencing of β2 adrenergic receptor (*ADRB2*) does not abolish the protective effects of formoterol and salbutamol against OA-induced cytotoxicity. (**A**) Relative *ADRB2* mRNA expression in PC-12 cells transfected with *ADRB2* siRNA confirms efficient knockdown compared to non-transfected control. PCR data were analyzed using the ΔΔCt method having *β-actin* as the housekeeping gene, while cells transfected with the negative control were used as calibrator. The bar graph represents the mean ΔΔCt value from 3 independent experiments. (**B**) Crystal violet assay showing cell viability in siRNA-transfected and non-transfected (WO siRNA) PC-12 cells treated with OA, with or without co-treatment with formoterol (FO, 10 μΜ) or salbutamol (SA, 10 μΜ) for 24 h. Data represent *n* = 3 independent experiments. Statistical analysis was performed using *t*-test for (**A**) and one-way ANOVA followed by Tukey’s multiple-comparison test for (**B**). ^#^ *p* < 0.05 versus control, ns: not significant.

To further examine whether protection depended on β2-AR expression, *ADRB2* was silenced using siRNA. *ADRB2* siRNA reduced *ADRB2* mRNA expression by approximately 80%, indicating substantial but incomplete knockdown (Figure 3A). In control, or cells where β2-AR was silenced with siRNA, OA reduced the viability to 60.3 ± 1.5% and 56.6 ± 0.7%, respectively. In the siRNA-treated cells, viability was 71.3 ± 2.0% and 71.4 ± 2.3% in FO- and SA-treated cells, respectively, while in the control cells without knockdown, viability was similar and amounted to 68.9 ± 1.5%, and 70.5 ± 2.8% for the FO- and SA-treated cells, respectively. Salbutamol and formoterol partially reversed the OA effects, and the results were similar in control and *ADRB2* siRNA-treated cells. These findings support the pharmacological blockage data, but because knockdown was validated only at the mRNA level, the siRNA data should be interpreted as supportive rather than definitive evidence of β2-AR independence.

Because the β2-AR blockade and knockdown experiments suggested that protection may involve mechanisms beyond canonical β2-AR activation, we next examined redox-related effects. Formoterol but not salbutamol showed antioxidant effects, based on the DPPH assay. As shown in Table 1, formoterol showed a concentration-dependent antioxidant effect, as the % DPPH activity was reduced from 100% to 79.0 ± 4.3%, 68.1 ± 4.5%, 71.8 ± 1.4%, and 59.8 ± 1.1% at 10, 25, 50 and 100 μM concentrations, respectively (*p* < 0.05 versus control). No statistical significance was observed at the 1 μM concentration. On the other hand, salbutamol at the same concentrations did not show any antioxidant effect. The positive control curcumin, a well-known antioxidant, had a very strong effect, resulting in 85.7 ± 1.5%, 80.1 ± 6.1%, 68.0 ± 2.1, 32.8 ± 1.0%, and 12.2 ± 0.8% remaining DPPH activity at the 1, 10, 25, 50 and 100 μM concentrations used (*p* < 0.05 vs. control). Compared to curcumin, the effect of formoterol was weaker, yet it showed a clear antioxidant effect. As the formoterol response was modest and not fully monotonic across concentrations, the DPPH data should be interpreted cautiously as evidence of limited direct radical-scavenging activity.

**Figure 4:**
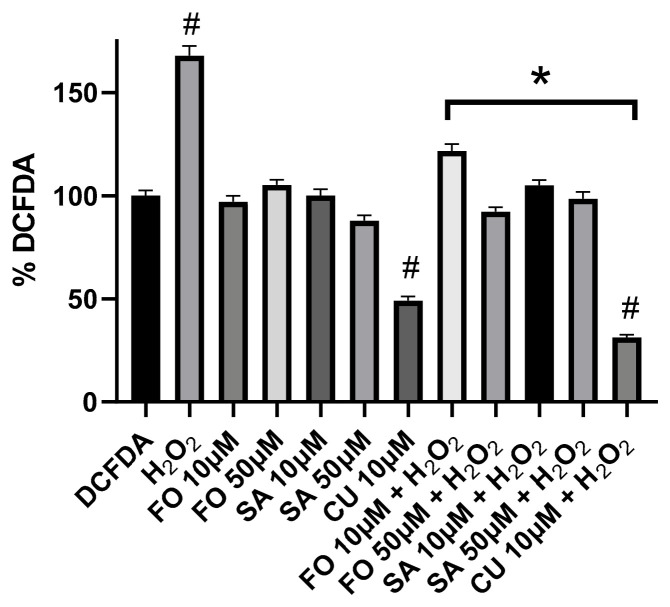
Intracellular assessment of the antioxidant activity of formoterol and salbutamol using DCFDA assay. (**A**) PC-12 cells were loaded with DCFDA and then treated for 1 h with H_2_O_2_ (500 µM), alone or in combination with formoterol (FO; 10 or 50 µM), salbutamol (SA; 10 or 50 µM), or curcumin (CU; 10 µM). Curcumin was included as a positive antioxidant control. Data are presented as mean ± SEM from n = 3 independent experiments, with 3 technical replicate wells per condition per experiment. Statistical analysis was performed using one-way ANOVA followed by Tukey’s multiple-comparison test. * *p* < 0.05 vs. H_2_O_2_, ^#^ *p* < 0.05 versus DCFDA control.

We next evaluated the ability of salbutamol and formoterol to reduce intracellular ROS levels in PC-12 cells as a functional measure of antioxidant activity. ROS were generated by H_2_O_2_ treatment. As shown in Figure 4, treatment of cells with 500 μM H_2_O_2_ for 1 h induced ROS by 167.9 ± 4.7% compared to control cells (regarded as 100%). In cells treated with formoterol, ROS generation was reduced to 121.6% ± 3.4%, and 92.3% ± 2.1% for the 10 and 50 μM formoterol concentrations, respectively, and for salbutamol, the ROS generation was reduced to 105.1 ± 2.4% and 98.5 ± 3.4% for the 10 and 50 μΜ concentrations, respectively (*p* < 0.05). Curcumin, on the other hand, was a very strong antioxidant and even reduced baseline ROS to 31.2%, (*p* < 0.05 vs. control). Together, the DPPH and DCFDA findings suggest that formoterol may have some direct radical-scavenging capacity, whereas salbutamol may reduce intracellular ROS-associated fluorescence through indirect or context-dependent cellular mechanisms rather than direct radical scavenging.

**Figure 5:**
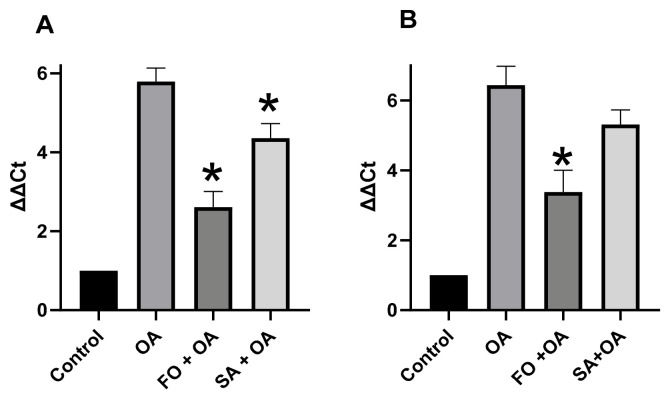
Effects of formoterol and salbutamol on pro-apoptotic genes in OA-induced cytotoxicity in PC-12 cells. mRNA levels of *Bax* (**A**) and *Bak* (**B**) were quantified by qPCR, normalized to *β-actin* and analyzed using the ΔΔCt method. OA treatment significantly upregulated both *Bax* and *Bak* expression compared to control. Co-treatment with formoterol (FO) substantially attenuated OA-induced increase, whereas salbutamol (SA) also reduced expression levels, but to a lesser extent. These data reflect mRNA expression of selected pro-apoptotic transcripts and do not establish functional inhibition of apoptosis. Data are presented as mean ± SEM from *n* = 4 independent experiments, with 3 technical replicate wells per condition per experiment. Statistical analysis was performed using one-way ANOVA followed by Tukey’s multiple-comparison test. * *p* < 0.05 compared to OA.

Finally, we examined whether salbutamol and formoterol altered OA-induced expression of the pro-apoptotic transcripts. As shown in Figure 5, OA induced pro-apoptotic genes *Bax* and *Bak* by 5.8-fold and 6.4-fold, respectively, and formoterol inhibited the induction of *Bax* and *Bak* compared to OA-only-treated cells. Specifically, the fold change relative to untreated control was reduced from 5.8 to 2.6 in the formoterol-treated cells (10 μΜ) for *Bax*, and from 6.4 to 3.4 for *Bak* (*p* < 0.05). For salbutamol (10 μΜ), the fold change relative to untreated control was reduced from 5.8 to 4.4 for *Bax*, and from 6.4 to 5.3 for *Bak*, respectively; however, statistical significance was achieved only for *Bax*. These qPCR data indicate reduced expression of selected pro-apoptotic transcripts but do not establish functional inhibition of apoptosis. Confirmation would require additional assays such as cleaved caspase-3, caspase-3/7 activity, Annexin V/propidium iodide staining, mitochondrial membrane potential analysis, or BAX/BAK protein measurement.

## 4. Discussion

This study examined whether salbutamol and formoterol attenuate OA-induced cytotoxicity in undifferentiated PC-12 cells. Both compounds improved cell viability in crystal violet and MTT assays, although the magnitude of protection differed between assays. These results support cytoprotective activity in this acute PC-12/OA model, but they do not establish disease-modifying activity in Alzheimer’s disease. The difference between crystal violet and MTT outcomes is important. Crystal violet staining primarily reflects adherent cell biomass and cell number, whereas MTT reduction reflects mitochondrial metabolic activity. The stronger recovery observed in the MTT assay may indicate that salbutamol and formoterol preserve mitochondrial-reducing capacity more effectively than total adherent cell biomass, or it may reflect assay-specific sensitivity.

A central, though provisional, observation of this study is that protection by formoterol and salbutamol persisted after β2-AR blockage with ICI-118,551 and after *ADRB2* mRNA knockdown. These results are consistent with a β2-AR-independent protective component in this PC-12/OA model. However, they do not establish receptor independence, since β2-AR protein expression and receptor-dependent signaling were not measured, and thus the evidence remains incomplete. Residual receptor protein, incomplete knockdown, compensatory signaling, or non-canonical β2-AR signaling cannot be excluded. As previously discussed in a review [13], evidence on the role of β2-AR activation is controversial, with some studies showing protective and some negative effects. In support of protective effects, studies show that β2-AR activation restores lysosomal function and autophagy; promotes degradation of Aβ; enhances neurogenesis, synaptic health, and cognitive function; reduces amyloid plaques, mitigates Aβ-induced inflammation and synaptotoxicity; and decreases neuroinflammatory responses. Conversely, other studies show that β2-AR activation may lead to impaired neurotransmitter signaling, disrupt learning and memory, enhance γ-secretase, promote amyloid plaques, and contribute to tau hyperphosphorylation and neurodegeneration. Our results suggest that SA and FO retain protective effects under conditions of β2-AR blockage or reduced *ADRB2* mRNA expression, supporting the possibility of a β2-AR-independent protective component. The results add to the existing knowledge of the protective effects of β2-AR agonists, and support further mechanistic investigation of both compound-specific effects. Future studies should confirm β2-AR knockdown at the protein level, assess receptor-dependent cAMP responses, and confirm functional ICI-118,551 activity in this system.

The antioxidant data suggests that formoterol and salbutamol may influence cellular oxidative stress through different mechanisms. Formoterol showed modest direct radical-scavenging activity in the cell-free DPPH assay, whereas salbutamol did not. In contrast, both compounds reduced H_2_O_2_-induced DCFDA fluorescence in cells. Thus, reduced DCFDA signal should not be interpreted as proof that both compounds directly scavenge free radicals. For salbutamol in particular, the DCFDA effect may reflect indirect modulation of cellular redox pathways, mitochondrial ROS generation, or other cellular responses.

Our results are in line with findings from the literature, as tested in a number of different cell types. Formoterol and salbutamol have been reported to limit ROS formation and protect cells from oxidative stress [15,16]; and in neutrophils, formoterol showed a stronger antioxidant effect than salbutamol [17], in line with our results. In a hypoxia/reoxygenation model, formoterol not only reduced endothelial cell death but also lowered intracellular ROS levels [16]. These antioxidant effects appear to lie outside the classic bronchodilatory pathway and contribute to the broader cytoprotective profile of β2-agonists.

Evidence for β2-agonists’ anti-apoptotic activity is mixed, but the activity seems to be mediated by the β2 receptors. Protective effects have been described in endothelial cells [16], airway eosinophils [18] and cardiac myocytes, where β2-receptor stimulation delivers a Gi-dependent signal via PI3K [19]. Conversely, salbutamol and formoterol induced apoptosis in B-chronic lymphocytic leukemia cells through a β2-receptor-independent mechanism [20]. In skeletal muscle and cancer cachexia models, formoterol decreased proteolysis and apoptosis, thereby exerting anti-catabolic effects [21]. Additional work has shown prolonged eosinophil survival via the cAMP–protein kinase A pathway [22]. Our data showed reduced OA-induced *Bax* and *Bak* transcript expression, particularly after formoterol treatment, but they do not demonstrate functional inhibition of apoptosis. Protein-level and functional apoptosis assays are required to determine whether these transcript changes correspond to reductions in apoptotic cell death.

The neuroprotective effects of SA and FO have also been documented. Salbutamol increased survival motor neuron protein in spinal muscular atrophy cells by limiting ubiquitin-mediated degradation [23]. Formoterol attenuated mitochondrial dysfunction in a Parkinson’s disease model, apparently by activating mitophagy and related signaling pathways [24]. These results suggest possible neuroprotective benefits.

Importantly, SA, but not FO, has been shown to directly inhibit tau-filament formation in vitro in a cell-free system [11]. Formoterol has been shown to activate the Akt–mTOR pathway in skeletal muscle [25], a signaling axis linked to tau phosphorylation control. These studies provide useful direction for future mechanistic work to delineate the mechanism of protection offered by FO and SA in OA-induced cell death in PC-12 cells, since OA is able to induce tau hyperphosphorylation.

The use of OA is both useful and limited. OA induces phosphatase inhibition, tau-related stress, oxidative injury [26], and cytotoxicity, but OA exposure is an acute pharmacological insult and does not capture the full biology of Alzheimer’s disease. The model does not include chronic tau pathology; amyloid pathology; neuroinflammation; aging; vascular effects; or interactions among neurons, astrocytes, and microglia. In addition, this study did not directly measure tau phosphorylation. Future studies should measure total tau and phosphorylated tau to determine whether salbutamol or formoterol modify OA-induced tau-related changes.

Our findings, though not correlated with a defined mechanism of action, are partly supported by clinical association data. A recent cohort study suggested that selective β2-AR agonists are associated with a decreased risk of developing AD, while non-selective AR antagonists are associated with an increased risk [7].

The translational relevance of these findings remains uncertain. The study was performed in undifferentiated PC-12 cells using an acute OA insult, and the active concentrations used in several assays may not reflect achievable CNS exposure in vivo. The 0.1 to 10 µM concentrations used in viability assays, and the 50 µM concentration used in DCFDA experiments are useful for in vitro mechanistic screening, but they may not reflect achievable free concentrations in the central nervous system in vivo. Therefore, these findings do not establish clinical feasibility, and the data should be interpreted as preliminary in vitro evidence of cytoprotection rather than evidence supporting clinical repurposing. Future work should define the mechanism of protection; validate receptor-independent activity at the protein and functional levels; assess tau phosphorylation; and test more disease-relevant systems, such as differentiated PC-12 cells, primary neurons, human induced pluripotent stem cell-derived neurons, and in vivo models. The concentrations used in this study also require cautious interpretation.

In conclusion, salbutamol and formoterol attenuated OA-induced cytotoxicity in undifferentiated PC-12 cells. Protection was retained after ICI-118,551 treatment and *ADRB2* mRNA knockdown, suggesting a potential β2-AR-independent protective component, while definitive exclusion of β2-AR involvement requires protein-level and functional validation. Formoterol showed modest direct radical-scavenging activity and reduced OA-induced *Bax* and *Bak* mRNA expression, whereas salbutamol reduced intracellular ROS-associated fluorescence and *Bax* mRNA expression without detectable DPPH activity. Overall, these findings provide preliminary in vitro evidence that selected β2-agonists can reduce OA-induced cytotoxicity through mechanisms that may extend beyond classical β2-AR activation.

## Figures and Tables

**Table 1:** Antioxidant activity of formoterol and salbutamol using DPPH assay. Cell-free DPPH radical-scavenging assay using formoterol, salbutamol, or curcumin at 1, 10, 25, 50, and 100 µM. Values represent percentage of the DPPH-only control after blank correction; lower values indicate greater radical-scavenging activity. Data are presented as mean ± SEM from *n* = 3 independent experiments, with 3 technical replicate wells per condition per experiment. Statistical analysis was performed using one-way ANOVA followed by Tukey’s multiple-comparison test. * *p* < 0.05 versus control.

Treatment	% DPPH Activity
Control	100 ± 2.1
CU 1 μΜ	85.7 ± 1.5 *
CU 10 μΜ	80.1 ± 6.1 *
CU 25 μΜ	68.0 ± 2.1 *
CU 50 μΜ	32.8 ± 1.0 *
CU 100 μΜ	12.2 ± 0.8 *
FO 1 μΜ	90.9 ± 3.3
FO 10 μΜ	79.0 ± 4.3 *
FO 25 μΜ	68.0 ± 4.5 *
FO 50 μΜ	71.8 ± 1.4 *
FO 100 μΜ	59.8 ± 1.1 *
SA 1 μΜ	100.6 ± 4.9
SA 10 μΜ	98.5 ± 4.0
SA 25 μΜ	94.6 ± 1.6
SA 50 μΜ	99.7 ± 1.4
SA 100 μΜ	99.6 ± 0.8

## Data Availability

All data supporting the findings of this study are available within the paper.
